# Modulatory effects of photobiomodulation combined with resistance exercise on lymph node response during rheumatoid arthritis induction in Wistar rats

**DOI:** 10.31744/einstein_journal/2026AO2060

**Published:** 2026-07-22

**Authors:** Aline Reginato, Rafael Andrade Menolli, Rose Meire Costa, Lucinéia de Fátima Chasko Ribeiro, Gladson Ricardo Flor Bertolini

**Affiliations:** 1 Universidade Estadual do Oeste do Paraná Cascavel PR Brazil Universidade Estadual do Oeste do Paraná, Cascavel, PR, Brazil.; 2 Universidade Estadual do Oeste do Paraná Cascavel PR Brazil Postgraduate Program in Biosciences and Health, Universidade Estadual do Oeste do Paraná, Cascavel, PR, Brazil.

**Keywords:** Exercise, Rats, Wistar, Arthritis, rheumatoid, Low-level light therapy, Immune system, Lymphocytes, Arthritis

## Abstract

**Objective:**

This study aimed to evaluate the effects of resistance exercise associated with photobiomodulation on the popliteal lymph nodes of Wistar rats subjected to an acute-phase experimental model of rheumatoid arthritis.

**Methods:**

The study consisted of 64 male Wistar rats divided into eight groups (n=8), including control, physical exercise, and photobiomodulation groups. The arthritis groups were subjected to an induction protocol using Complete Freund's Adjuvant and underwent an acute inflammatory period of 7 days, during which they received resistance exercise and photobiomodulation according to their respective group assignments.

**Results:**

There was a significant increase in the total lymph node area of the groups subjected to rheumatoid arthritis induction. A reduction in the number of lymphocytes and plasma cells in the medullary cord, as well as macrophages in the medullary sinuses, was observed in the groups treated with both resistance exercise and photobiomodulation. Additionally, a reduction in lymphocytes and reticular cells was observed in the groups treated with photobiomodulation alone.

**Conclusion:**

Treatment with resistance exercise or photobiomodulation was effective in modulating the immune response in the popliteal lymph node during the acute stage of the disease; however, combined therapy did not provide additional benefits compared with either treatment alone.

## Highlights

Rheumatoid arthritis increases the popliteal lymph node area in rats.

Resistance exercise and photobiomodulation modulate immune cell populations.

Both therapies reduce plasma cells and inflammatory cell infiltration.

Combined therapy shows no additional benefits over isolated treatments.

## In Brief

This experimental study in Wistar rats with induced rheumatoid arthritis showed that resistance exercise and photobiomodulation modulate immune responses in popliteal lymph nodes by reducing inflammatory cell infiltration. However, combined therapy did not provide additional benefits compared to isolated treatment.

## Graphical abstract

**Figure:**
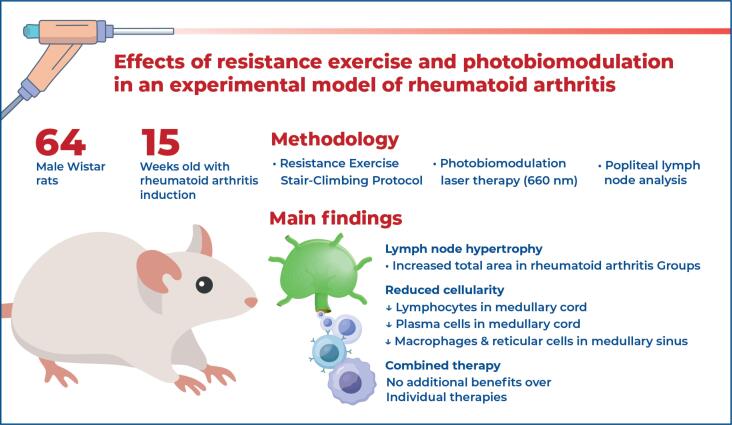

## INTRODUCTION

Rheumatoid arthritis (RA) is an inflammatory disease of unknown origin, classified as symmetrical polyarthritis, and is characterized by a cascade of inflammatory events affecting the synovial joints. ^(1)^ It arises from the interaction of genetic, epigenetic, and environmental factors, which promote crosstalk between the adaptive and innate immune systems, triggering the production of autoantibodies, mainly rheumatoid factors (RF), as well as the migration of T and B lymphocytes to the synovium and the intense activation of monocytes/macrophages in the affected tissues.^(2,3)^

Faced with these factors, the characteristic clinical symptoms of RA begin to manifest, including pain and edema, which progressively lead to movement limitations and functional disabilities, compromising the musculoskeletal, cardiovascular, and pulmonary systems and resulting in a decline in quality of life.^(4–6)^ These features reflect the systemic nature of RA, which explains the involvement of periarticular structures, including lymph nodes (LN). Lymph nodes are components of the lymphatic system responsible for mediating interactions between antigen-presenting cells and lymphocytes, thereby initiating adaptive immune responses and playing an integral role in the development, maintenance, and progression of aberrant immune responses.^(7–9)^

Lymph nodes have a complex structure that houses specialized immune cells within anatomically defined compartments with complementary functions. The cortical region, located beneath the capsule, is rich in lymphoid tissue, predominantly composed of B lymphocytes, which undergo maturation in germinal centers upon stimulation. The paracortical region contains a predominance of T lymphocytes. The medullary region is subdivided into two components: the medullary cords, composed mainly of lymphocytes, plasma cells, macrophages, and reticular cells; and the medullary sinuses, which are spaces between the cords containing lymph and cells originating from the cortical region, including reticular cells, lymphocytes, macrophages, and polymorphonuclear leukocytes. The medullary sinuses converge to form the efferent lymphatic vessels in the hilum region.^(8,10)^

Animal experiments using RA induction models are important for evaluating the structural and functional changes promoted by the disease, as well as for identifying effective treatments and strategies for symptom relief.^(11,12)^ One such model is arthritis induced by Complete Freund's adjuvant (CFA), which consists of a mannitol monooleate compound containing attenuated mycobacterium (Mycobacterium butyricum or *Mycobacterium* tuberculosis). This model reproduces key features of human RA, including histopathological changes, cell infiltration, hypersensitivity, and edema in the affected joints.^(11)^

The treatment of RA usually begins in the acute phase and aims to relieve pain, reduce edema, restore joint mobility, and improve overall function through conservative approaches, including patient education, pharmacological therapy, and physiotherapy.^(1,2,13,14)^ Although there is no gold standard treatment, first-line therapy typically involves the use of disease-modifying antirheumatic drugs (DMARDs), with methotrexate (MTX) widely considered the cornerstone treatment.^(15)^ It is also important to consider complementary approaches: for example, Gomes et al.^(11)^ demonstrated that physical exercise reduced nociception, edema, and leukocyte migration in an experimental RA model.

Hsieh et al.^(16)^ demonstrated that low-intensity laser therapy, a form of photobiomodulation (PBM), is effective in reducing inflammation and decreasing the number of inflammatory mediators and cells in a CFA-induced RA model. In this context, studies on non-pharmacological approaches, such as physical exercise and complementary therapies, have gained increasing relevance, as they minimize RA symptoms and promote improvements in cardiorespiratory fitness and cardiovascular health, increased muscle mass, reduced adiposity, and the maintenance of functional capacity.^(17–19)^

Given the systemic effects of RA, it is important to investigate the therapeutic effects of different modalities on tissues beyond the joints, such as nearby lymph nodes, which play a key role in immune responses, as well as to determine whether these treatments are effective in modifying the characteristics of non-articular tissues.

## OBJECTIVE

This study aimed to evaluate the histomorphometric effects of resistance exercise combined with photobiomodulation on the popliteal lymph nodes of Wistar rats subjected to an experimental model of rheumatoid arthritis.

## METHODS

This study was designed as a randomized experimental study and included 64 male Wistar rats, aged 15 weeks, with a body mass of 250±19g. The animals were housed at the Laboratory for the Study of Injuries and Physiotherapeutic Resources in polypropylene cages, with free access to water and food (ad libitum), under controlled temperature (22°C), and a 12-hour light/dark cycle. The study was approved by the Animal Use Ethics Committee of Unioeste (Protocol 19-19). All procedures followed the Animal Research: Reporting *In Vivo* Experiments (ARRIVE) ^(20)^ guidelines.

Animals were divided into eight groups: Control Group (CG, n=8): consisting of animals that were neither subjected to RA induction nor treated; Arthritis Group (AG, n=8): consisting of animals subjected to RA induction without treatment, PBM Control Group (PCG, n=8): consisting of non arthritic animals treated with photobiomodulation; PBM Arthritis Group (PAG, n=8): consisting of animals subjected to RA induction and treated with photobiomodulation; Exercise Control Group (ECG, n=8): - consisting of non-arthritic animals treated with resistance exercise; Exercise Arthritis Group (EAG, n=8): consisting of animals subjected to RA induction and treated with resistance exercise; PBM + Exercise Control Group (PECG, n=8): consisting of non-arthritic animals - treated with both therapies; and PBM + Exercise Arthritis Group (PAEG, n=8): consisting of animals subjected to RA induction and treated with both photobiomodulation and resistance exercise.

### Rheumatoid arthritis induction protocol

The rheumatoid arthritis model was induced as described by Gomes et al.^(21)^ and consisted of two 50μL injections of Complete Freund's Adjuvant (CFA Mycobacterium butyricum, 0.5mg/ml Difco®). The first injection was administered intradermally at the base of the tail for initial immunization, and seven days later, the second injection was administered into the tibiofemoral joint of the animals’ right hind limb. Animals not allocated to the lesion groups received sodium chloride injections (0.9%, Aster®) at the same time points and anatomical sites. The animals underwent an acute inflammatory period of 7 days, during which they either received or did not receive treatment according to their respective groups, after which they were euthanized.

### Treatment protocol

The treatment administered to the ECG, EAG, PECG, and PAEG Groups consisted of an adapted stair-climbing resistance exercise protocol.^(22)^ A vertical wooden ladder with 67 iron steps inclined at 60° was used. A resting chamber was positioned at the top of the ladder to allow the animals to recover between sets. The exercise protocol consisted of four sets of five climbs, with a 60-second interval between sets, using a 100 g overload attached to the tail, beginning 24 h after the intra-articular injection. ^(12)^ The animals underwent treatment for four consecutive days.

The PBM treatment consisted of four applications administered to the PCG, PAG, PECG, and PAEG. Animals in the remaining Groups received contact with the probe in the knee region of the right hind limb, and without light emission. The protocol involved PBM application at four sites: anterior to the patella, medial to the tibiofemoral joint, lateral to the tibiofemoral joint, and posterior to the popliteal region. The following parameters were used: point application technique, four application points, wavelength of 660nm, power of 30mW, spot area: 0.06cm^2^, energy density: 5 J/cm^2^ per point, irradiation time of 10 seconds per point, and total energy of 0.003 J per point.^(12)^ The equipment output power was verified before treatment.

### Euthanasia and lymph node processing

After 7 days of treatment, all animals were anesthetized with intraperitoneal injections of ketamine hydrochloride and xylazine. Following confirmation of the anesthetic state, euthanasia was performed by guillotine decapitation. The right popliteal lymph node was then resected and fixed in methacarn solution (70% methanol, 20% chloroform, and 10% glacial acetic acid). Subsequently, the lymph nodes were processed and embedded in paraffin in a " horizontal position to allow longitudinal sectioning. Sections of 5 μm thickness were obtained using an Olympus CUT 4055 microtome, and the slides were stained with hematoxylin and eosin for morphological and morphometric analyses.

Photomicrographs were obtained at magnifications of 40x and 400×. In the 40x images, the total area and medullary areas were measured using Image ProPlus 6.0 software to determine the cortical and medullary proportions of the lymph node. At 400x magnification, five visual fields of the medullary region were photomicrographed. The Grid Mask function of Image Pro Plus 6.0 using- 50 x 50μm squares, was applied to perform cell counts in two selected areas of each image: one corresponding to the medullary cord region and the other to the medullary sinus. This resulted in five counts for the medullary cord and five for the medullary sinus per animal. Lymphocytes and plasma cells were quantified in the medullary cords, whereas lymphocytes, macrophages, and reticular cells were quantified in the medullary sinuses. All analyses were performed in a blinded manner.

### Statistical analysis

For a large effect size (0.55), seven groups, α=0.05, and a statistical power of 0.85, a total of 63 animals was required; however, to facilitate equal group allocation, 64 animals were included. The data were analyzed using the SPSS statistical package (version 20.0). Initially, normality (Shapiro-Wilk test) and homoscedasticity (Levene's test) were assessed. Subsequently, the results were analyzed using two-way analysis of variance (ANOVA), followed by Tukey-HSD post-test. Statistical significance was set at p<0.05.

## RESULTS

**Figure 1:**
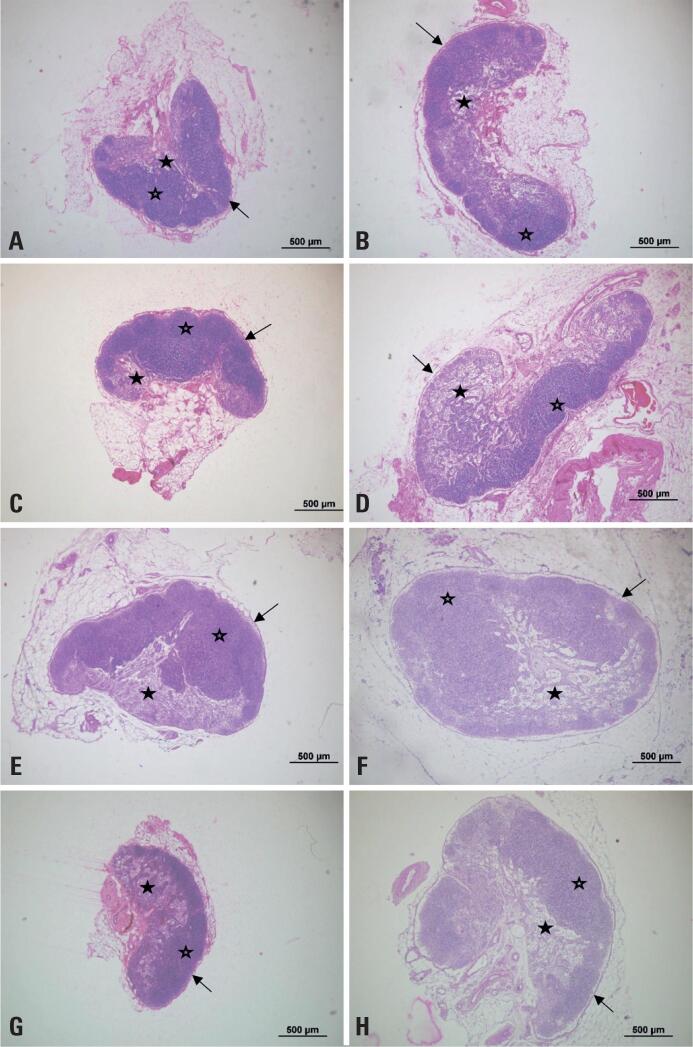
Photomicrographs of the total area of the popliteal lymph nodes of Wistar rats at 40x magnification. Hematoxylin and eosin staining. GC (A), GA (B), GCL (C), GCE (D), GCLE (E), GAE (F), GAL (G), and GALE (H). Cortical region (open star), medullary region (filled star), and connective tissue capsule (arrow)

Morphological analysis of the popliteal lymph nodes (Figure 1) showed that all groups presented normal anatomical characteristics, including a kidney or bean shape, morphology, surrounded by an intact dense connective tissue capsule, and a subcapsular space between the capsule and cortex, visible along almost the entire length of the organ. The cortical and medullary regions, as well as their respective structures, could be clearly distinguished.

**Figure 2:**
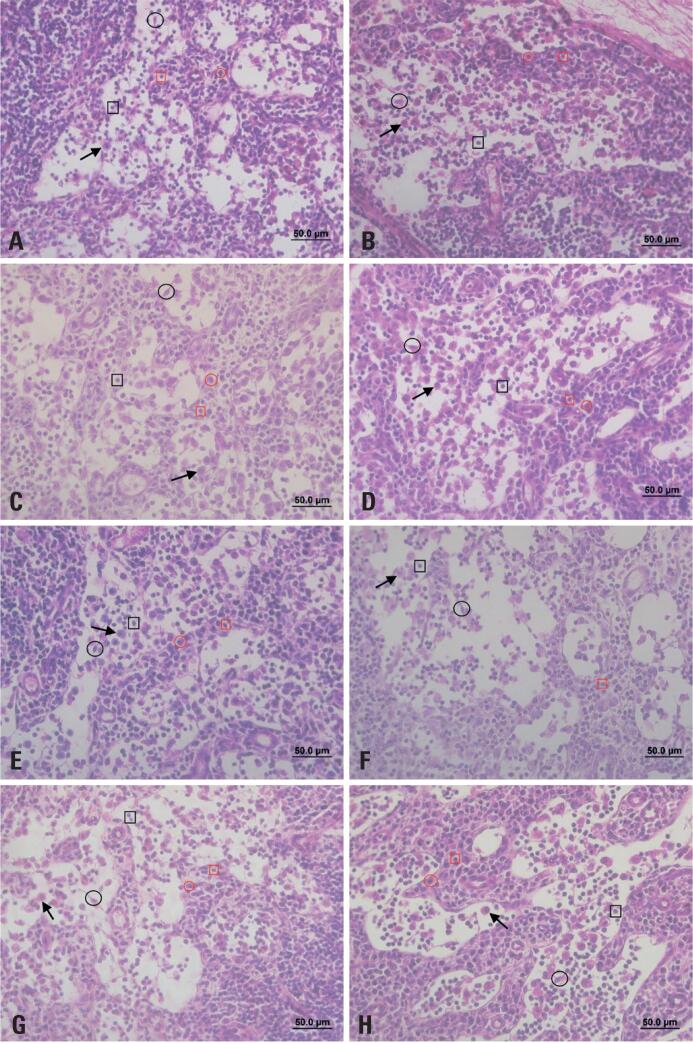
Photomicrographs of the medullary region of the popliteal lymph nodes of a Wistar rat at 400x magnification. Hematoxylin and eosin staining. GC (A), GA (B), GCL (C), GCE (D), GCLE (E), GAE (F), GAL (G), GALE (H). Lymphocyte in the medullary cord (red square), plasma cell in the medullary cord (red circle), macrophage in the medullary sinus (arrow), reticular cell in the medullary sinus (black circle), and lymphocyte in the medullary sinus (black square)

Regarding the medullary region (Figure 2), the medullary cords and sinuses could be identified in all groups. In general, the medullary cords exhibited greater cellularity than the medullary sinuses; however, in some animals from the AG, ECG, EAG, and PAEG Groups, a high number of cells was also observed within the medullary sinuses. The predominant cell population in all groups consisted of lymphocytes, followed by macrophages, whereas plasma cells and reticular cells were observed less frequently.

Analysis of the total lymph node area (Table 1) revealed a significant difference between the PAEG Group and the CG, ECG, PCG, PAG, and PECG Groups (p<0.05). For the same parameter, the PECG Group also differed significantly from the AG, EAG, and PAEG Groups (p<0.05), whereas the PCG Group differed from the AG, EAG, and PAEG Groups (p<0.05). These findings indicate that PBM alone or combined with exercise in control animals did not induce the changes in total lymph node area observed following arthritis induction. No significant differences were observed among the Groups regarding the percentage of the total area corresponding to the medulla or cortex (p>0.05) (Table 1).

**Table 1:** Histomorphometric data for total lymph node area and percentages of medullary and cortical areas

	Total area	% medula	% cortex
CG	2761853.2^BCD^	22.7^±^4^A^	77.2±4^A^
AG	3587244.2^AD^	35.5^±^4^A^	64.4±4^A^
ECG	2577363.6^BCD^	37.8^±^4^A^	62.1±4^A^
EAG	3535725.8^AD^	26.5^±^4^A^	73.4±4^A^
PCG	1916433.4^BC^	35.3^±^4^A^	64.6±4^A^
PAG	2535754.6^BCD^	33.3^±^4^A^	66.6±4^A^
PECG	1880904.2^BC^	37.8^±^4^A^	62.1±4^A^
PAEG	5587464.5^A^	32.6±5^A^	67.3±5^A^

Data expressed as mean ± standard deviation. Identical letters indicate no significant differences between groups. Histomorphometric analysis of total lymph node area, and percentages of medullary and cortical areas. A significant difference among groups was observed fortotal area (p=0.002). Used Data were analyzed using two-way ANOVA followed by Tukey-HSD post hoc test.

In terms of the number of lymphocytes in the medullary cord, the AG Group showed significantly higher values compared with the PAG, ECG, and PCG Groups (p<0.05). For the same parameter, the PCG Group differed significantly from the AG, PAEG, and PECG Groups (p<0.05), while the PAEG Group differed from the PCG and ECG Groups (p<0.05). These findings suggest that arthritis increased lymphocyte counts in the medullary cord, whereas PBM alone promoted changes in this paramenter. Regarding plasma cell counts in the medullary cord, the GC Group presented significantly higher valuescompared with the other groups, as did the AG Group in comparison with all remaining groups (p<0.05). In addition, the EAG Group showed a significant difference compared with the CG, AG, ECG, PAG, PECG, and PAEG Groups (p<0.05), indicating an effect of physical exercise on this variable (Table 2).

**Table 2:** Histomorphometric data for lymphocyte and plasma cell counts in the medullary cords

	Lymphocytes	Plasmocytes
CG	141.2±14^ABCD^	57.6±5^D^
AG	164.8±16^A^	40.4±3^A^
ECG	110.8±11^CD^	19.8±1^C^
EAG	129±13^ABCD^	13.6±1^B^
PCG	109±11^D^	16.4±1^BC^
PAG	121.8±12^BCD^	20±1^C^
PECG	147±14^ABC^	21±1^C^
PAEG	153.4±15^AB^	18.2±1^C^

Data expressed as mean±standard deviation. Identical letters indicate no significant differences between groups. Histomorphometric analysis of lymphocytes and plasma cells counts, in the medullary cord of the lymph node. Significant differences among groups were observed for the number of lymphocytes (p=0.027) and plasma cells (p<0.001). Data were analyzed using two-way ANOVA followed by Tukey-HSD post hoc test.

In terms of the number of lymphocytes in the medullary sinus (Table 3), the PCG Group showed a significant difference compared with all other Groups (p<0.05); In addition, the CG and PAG Groups differed significantly from the AG, EAG, PAEG, ECG, and PCG Groups (p<0.05), again suggesting effects of PBM, even in the absence of arthritis induction. For macrophage counts in the medullary sinus, the AG Group presented significantly higher values than the other groups (p<0.05), except for the CG Group (p>0.05). The CG Group also showed significant differences compared with the ECG, EAG, PCG, PAG, and PAEG Groups (p<0.05).

**Table 3:** Histomorphometric data for lymphocytes, macrophages and reticular cells counts in the medullary sinuses of the lymph nodes

	Lymphocytes	Macrophages	Reticular cells
CG	72.8±7^B^	49^±^4^AB^	40±2^A^
AG	134.6±13^C^	60±6^A^	40.8±2^A^
ECG	103.8±10^CD^	34.4±3^DEF^	37.8±2^ABD^
EAG	115±12^CD^	30±3^CD^	27.7±3^CD^
PCG	51.2±5^A^	27.6±3^CF^	27±2^CE^
PAG	72.2±7^B^	24.6±2^C^	20.2±2^C^
PECG	95.6±9^BD^	40.4±4^BD^	50±2^F^
PAEG	115.6±11^CD^	26±2^CE^	31.8±2^BDE^

Data are expressed as mean±standard deviation. Identical letters indicate no significant differences between groups. Histomorphometric analysis of lymphocytes, macrophages, and reticular cells counts in the medullary sinuses of the lymph nodes. Significant differences among groups were observed for the number of lymphocytes (p<0.001), macrophages (p<0.001) and reticular cells (p<0.001). Data were analyzed using two-way ANOVA followed by Tukey's HSD post hoc test.

For the same parameter, the PECG Group showed a significant difference compared with the AG, PCG, PAG, and PAEG Groups (p<0.05), while the PAG Group differed significantly from the CG, AG, ECG, and PECG Groups (p<0.05). Regarding the number of reticular cells in the medullary sinus, the PECG Group showed significant differences compared with all other groups (p<0.05). The CG Group also differed significantly from the PCG, PAG, EAG, PECG and PAEG Groups (p<0.05). In addition, the PAG Group presented a significantly lower number of reticular cells than the CG, AG, ECG, PECG, and PAEG Groups (p<0.05), indicating opposite effects between the two therapies (Table 3).

## DISCUSSION

This study aimed to evaluate the histomorphometric effects of resistance exercise combined with photobiomodulation (PBM) on the popliteal lymph nodes of Wistar rats subjected to an experimental model of RA. The groups, subjected to RA induction exhibited an increase in total lymph node area. Additionally, the number of lymphocytes in the medullary cords was lower in the groups treated with either resistance exercise or PBM alone. Plasma cell counts were reduced in the groups subjected to both isolated and combined therapies. In the medullary sinuses, the PBM-treated groups presented lymphocyte counts similar to those of the Control Group (CG), whereas macrophage and reticular cell counts were reduced in the groups treated with either resistance exercise or PBM alone.

Lymph nodes are integral components of the lymphatic system and play an essential role in immune responses to antigens present in the body.^(23–25)^ One of the main indicators of reactivity is lymph node hypertrophy, which occurs as a result of increased cell activation. In the present study, changes in total lymph node area were observed, with the RA-induced groups exhibiting larger areas compared to the Control Groups, indicating heightened reactivity of the lymphatic system in response to the experimental model. However, the absence of alterations in the cortical-to-medullary ratio may be explained by the findings of Benaglio et al.,^(8)^ who reported that activation of the lymphatic system promotes l hypertrophy of the stromal tissue in both the cortical and medullary regions. This process facilitates the migration of mature dendritic cells from peripheral tissues through the afferent lymphatics, enhances lymphocyte recruitment from the bloodstream, and transiently reduces leukocyte efflux through the efferent lymphatics.

The ability to specifically recognize foreign molecules - and coordinate immune responses is a key function of mature B lymphocytes (BLs) and T lymphocytes (TLs). Activation of BLs initiates cellular proliferation and differentiation, resulting in the generation of plasma cells that produce immunoglobulins with high affinity for the antigenic epitope responsible for activation. These cells ultimately differentiate into memory BLs, and play an important role in the production of rheumatoid factor and anti-citrullinated peptide antibodies, both of which are involved in RA pathogenesis. In addition, BLs present antigens to T cells, thereby perpetuating the autoimmune response, and secrete TNF-α and IL-6, contributing to the maintenance of the inflammatory environment.^(26,27)^ The primary function of TL is to destroy cells presenting foreign molecules on their surface. In addition, TLs synthesize pro-inflammatory cytokines that activate BLs and recruit additional phagocytic cells, such as macrophages. The CD4+ subpopulation plays a crucial role in the inflammatory process, particularly through its differentiation into Th1 and Th17 cells. Th1 cells secrete IFN-γ and TNF-α, whereas Th17 cells release several interleukins, including IL-6, IL-8, IL-17A, IL-17F, IL-21, and IL-22, as well as metalloproteinases that recruit neutrophils and stimulate RANKL expression, all of which are directly associated with inflammation and bone destruction. Regulatory T cells are also involved however, evidence suggests an imbalance between regulatory and pro-inflammatory T-cell populations in RA, resulting in impaired immune tolerance.^(27)^ Macrophages are highly efficient phagocytic cells responsible for clearing a wide range of nanoparticles, bacteria, and apoptotic cells. Within the lymphatic system, macrophages located in the medullary sinuses of the lymph nodes remain in direct contact with the lymphatic fluid. Equipped with multiple pattern recognition receptors, these cells act as a "flypaper," capturing and retaining pathogens, and thereby preventing the systemic spread of infection. Macrophages are also major sources of inflammatory cytokines in RA, particularly TNF-α, which is considered a key cytokine in the inflammatory cascade, as it induces the production of other cytokines such as IL-1β and IL-6.^(28–30)^ According to Lin et al.,^(2)^ lymphatic activity in mice subjected to an RA model occurs in two phases: an expansion phase, corresponding to the period of peak reactivity, and a collapse phase, during which the lymph node returns to its baseline state. During the expansion phase, there is an accumulation and migration of B and T cells in both the cortical and medullary regions, with a predominance of T cells in the medullary region. These findings support the results of the present study, in which the RA Group (AG) exhibited a significantly higher number of lymphocytes in the medullary sinus compared with the Control Group (CG).

Physical exercise can elicit acute or chronic biochemical and physiological responses depending on its frequency, volume, and intensity. These responses may enhance immune function by promoting the transient redistribution of immune cells to peripheral tissues, resulting in increased immunocompetence and an attenuation of inflammatory diseases. Conversely, exercise performed below a minimum intensity threshold does not substantially affect body homeostasis and therefore fails to induce significant biochemical or physiological adaptations.^(31,32)^ Damasceno et al.^(33)^ investigated different physical training protocols in an induced RA model and found that all protocols promoted increased inflammatory cell influx and upregulation of anti-inflammatory gene expression. These findings are consistent with the results of the present study, which demonstrated an increased number of lymphocytes in the medullary sinus of the groups subjected to the resistance exercise protocol compared with the control and PBM Groups.

Additionally, the groups subjected to the resistance exercise protocol exhibited a reduction in the number of macrophages and reticular cells in the medullary sinuses, as well as a decrease in plasma cell counts in the medullary cords.

Photobiomodulation exhibits biophysical and biological effects mediated by the absorption of red and infrared radiation by chromophores, which are components of the mitochondrial respiratory chain. The absorption of this energy triggers a cascade of biochemical events, leading to increased enzymatic activity, ATP production, protein synthesis, cell proliferation, and collagen deposition. ^(34,35)^ In addition, PBM can modulate the inflammatory process in various conditions involving joint inflammation by influencing pro-inflammatory mediators. Previous studies have demonstrated that PBM reduces the expression of cytokines such as IL-1β, IL-6, and TNF-α, as well as decreases the number of inflammatory cells, including macrophages and neutrophils.^(16,35)^

Pallotta et al.^(36)^ investigated the effects of photobiomodulation using infrared radiation (810 nm) on experimentally induced knee inflammation in rats. Their study demonstrated a reduction in inflammatory signs and evaluated the role of apoptosis in the anti-inflammatory effects of different low-level laser therapy (LLLT) doses in an experimental model of RA. Similarly, Anjos et al.^(37)^ reported decreased levels of pro-inflammatory cytokines following LLLT treatment. These findings are consistent with the results of the present study, in which the groups treated with LLLT exhibited reduced cell counts in both the medullary cord and medullary sinuses compared with the other groups.

Neves et al.^(12)^ evaluated the effects of PBM combined with resistance exercise on nociception and leukocyte migration in an experimental model of RA, demonstrating positive effects on inflammatory modulation, reduced leukocyte migration, and improved functional recovery. Similar findings were observed in the present study, in which the combination of resistance exercise and PBM reduced the number of macrophages and plasma cells compared with the CG and AG Groups. However, the PEAG Group (resistance exercise and PBM) exhibited a higher lymphocyte count in both the medullary sinuses and medullary cords compared with the groups treated with either intervention alone. Additionally, the PECG Group, which underwent resistance exercise alone, exhibited a higher number of reticular cells than the other groups.

The immune system is highly responsive to exercise, and this response varies according to the intensity and duration of the activity, reflecting the degree of physiological stress imposed by the workload.^(31)^ Similarly, PBM produces different effects depending on parameters such as dose, technique, application site, and treatment duration.^(38,39)^ In the present study, the combination of exercise and PBM may have intensified the stimulus, resulting in an increased number of immune cells. However, a limitation of this study is the absence of interleukin analysis, which could have provided a more comprehensive understanding of the synergistic effects of these therapies. It should also be emphasized that pharmacological therapy remains the first-line treatment for PBM, and physical exercise should be considered a complementary approach that may contribute to favorable therapeutic results.

## CONCLUSION

In conclusion, both resistance exercise or photobiomodulation were effective in modulating the immune response in the popliteal lymph nodes during the acute stage of rheumatoid arthritis. However, the combined application of these therapies did not provide additional benefits compared with their isolated use.

## Data Availability

The data are in the possession of the authors and can be made available upon request.
